# Association between periodontitis and physical fitness in law enforcement workers

**DOI:** 10.1007/s00784-024-06135-x

**Published:** 2025-01-31

**Authors:** Jacopo Buti, F. Ronca, P. W. Burgess, J. Gallagher, P. Ashley, I. Needleman

**Affiliations:** 1 Unit of Periodontology, University College London, Eastman Dental Institute, London, UK; 2 Institute of Sport Exercise & Health, University College London, London, UK; 3 Institute of Cognitive Neuroscience, University College London, London, UK; 4 Unit of Paediatric Dentistry, UCL Eastman Dental Institute, London, UK; 5 University College London Centre for Sports Dentistry, London, UK

**Keywords:** Oral health, Periodontitis, Cardiorespiratory fitness and physical fitness

## Abstract

**Objectives:**

Oral and periodontal health have been linked to systemic health, cardiovascular disease and inflammation markers. Physical fitness has been linked to increased inflammatory response, but only few studies have investigated the association between oral health with physical activity. The aim of this study was to evaluate the association between oral and periodontal health status and physical fitness in British law enforcement workers.

**Methods:**

89 subjects were recruited between November and December 2019. Cardiopulmonary fitness was measured by Maximal Oxygen Uptake (VO_2max_) (ml/kg/min) and Maximum Load (Load_max_) (W) generated at the end of the Bruce incremental treadmill test; physical activity levels through accelerometers; functional strength tests by Countermovement Jump (CMJ) Power (W) and Height (cm) average. Oral variables included percentage of sites with PPD > 4 mm (% PPD > 4), full-mouth bleeding score (FMBS) and Decayed, Missing, and Filled Teeth (DMFT) index. Linear regression models were adjusted for age, gender and fat %.

**Results:**

Mean age was 41.5 years (range 23–61; 71.9% male). Higher % PPD > 4 was consistently correlated with lower Load_max_ (-4.96; *p* = 0.092), CMJ Height average (-0.39; *p* = 0.064), and press-ups in 60 s (-0.85; *p* = 0.052) though the associations were not statistically significant. FMBS was associated with the % of a day spent in sedentary (0.78; *p* = 0.030) and light activities (-0.75; *p* = 0.022).

**Conclusions:**

Periodontitis may be negatively associated with certain components of physical performance.

**Clinical relevance:**

Oral health status may be associated with physical fitness and functional strength.

## Introduction

Periodontitis is a chronic multifactorial inflammatory disease associated with dysbiotic plaque biofilms and characterized by progressive destruction of the tooth-supporting apparatus [1]. Periodontitis represents a major public health problem due to its high prevalence (45–50%) [2] and evidence supporting associations with several non‐communicable diseases (NCDs), including cardiovascular disease (CVD) and Type II diabetes [3–5]. Potential mechanisms include bacteraemia [6] and the associated systemic inflammatory sequelae, including elevations in C‐reactive protein and oxidative stress [7].

Physical fitness, encompassing both muscular and cardiorespiratory fitness [8], is strongly associated with overall morbidity and mortality in the general population [9]. Cardiorespiratory fitness (CRF) is defined as the ability of the cardiovascular and pulmonary system to deliver oxygen to sustain musculoskeletal function during exercise [10] and is strongly associated with performance in endurance activities, such as running and cycling. Absolute values of oxygen uptake and performance related to CRF are strongly influenced by age, gender and genetic factors, but are nonetheless regarded as accurate predictors of general health [11, 12], where CRF is an established predictor of prognosis in healthy subjects and of risk of mortality in clinical cohorts [13–15], and is more strongly associated with poor health than obesity or levels of physical activity [9, 16]. Muscle strength is also a key aspect of health and wellbeing [8] with international physical activity guidelines incorporating recommendations for minimum levels of activity focused on muscle strength [17].

To date, few studies have investigated the relationship between physical fitness and periodontal inflammation. The earliest evaluation was conducted in a Japanese population of 517 males and 113 females aged 23 to 83 years who were categorised by their worst community periodontal index of treatment needs (CPITN) finding as well as cycle ergometry for measuring Maximal Oxygen Uptake (VO_2max_). 67.9% of participants were recorded as CPITN 3 or above [18]. The VO_2_ at anaerobic threshold was reported as tending to be negatively related to CPITN scores although this was not statistically significant (*p* = 0.079). CPITN was also employed in a further study from Japan reporting on 1,160 individuals aged 20–77 years of whom 41.6% had at least one sextant with code 3 or 4. A statistically significant association (*p* = 0.02) was found between VO_2max_ and CPITN in the adjusted multiple regression model [19]. Interestingly, further investigation of the interaction of BMI and VO_2max_ with severe periodontitis (≥ 3 sextants with CPITN 3 or 4) demonstrated a statistically inverse relationship between CRF and severe periodontitis with low but not high BMI after adjustment for age, gender, number of teeth, smoking status, fasting plasma glucose and systolic blood pressure. In non-smoking sedentary males aged 45–65 years, moderate and severe periodontitis were statistically significantly associated with lower CRF measured via cycle ergometry [20]. In this study conducted in Germany, a full-mouth periodontal examination was conducted and 58% of the participants were classified as having moderate or severe periodontitis. Contradictory findings were reported from a secondary analysis of the NHANES databases (1999–2004) on 2,863 younger males and females aged 20–49 years who underwent partial-mouth periodontal examination and treadmill testing to measure VO_2max_. No statistically significant association were found between VO_2max_, attachment loss and probing depth although less than 5% of the sample had moderate-severe periodontitis [21]. Indirect evidence was reported in a study of 111 male military police officers aged 20–56 years undergoing standard physical fitness testing (PFT) that included measuring distance run in 12 min as well as strength-based testing and partial-mouth periodontal examination; 36% of the participants were reported as having at least moderate periodontitis. Multivariate regression analysis revealed statistically significant associations between the overall PFT and mean probing depth, attachment level, bleeding on probing, and having either probing depth ≥ 5 mm or attachment loss ≥ 4 mm in at least one tooth. Endodontic burden, which included both apical periodontitis and the presence of root treated teeth did not influence the outcomes [22, 23].

In summary, the relationship between periodontal health and physical fitness is unclear. Clarifying the relationship is important since periodontal ‘ill-health’ is common with some level of periodontitis in around half of the population and severe periodontitis affecting 11.2% of people worldwide [24]. Understanding the impact of periodontitis on physical fitness could inform potential mechanisms linking periodontitis and CVD and all-cause mortality. Assessing cardiorespiratory fitness objectively in the laboratory can be perceived as demanding by potential participants thereby resulting in selection bias with any study. In view of the periodic fitness testing of British law enforcement officers, this element was not anticipated to be an issue for recruitment. A limitation with several of the existing studies has been the use of unvalidated or inefficient measures of exposure and outcome including CPITN, partial mouth periodontal recording and physical fitness testing. We designed this study with collection of both validated full-mouth periodontal measures and validated measures of physical fitness (VO_2max_) and power (CMJ) both measured by researchers expert in their respective fields to give greater confidence in study findings. Therefore, the aim of this study was to evaluate the association between oral and periodontal health status and physical fitness in British law enforcement workers.

## Materials and methods

### Study design and setting

This cross-sectional study includes study participants originally recruited as part of a randomised controlled clinical trial to assess the effect of physical activity interventions in British law enforcement workers. British law enforcement workers were recruited between November and December 2019. Following baseline data collection, the trial was stopped due to the COVID-19 pandemic. Hence, data reported and analysed in the present study refer only to baseline assessment measures for this study population collected between January and March 2020.

Volunteers attended the Institute of Sport, Exercise and Health (ISEH) where anthropometric measurements were taken, and they underwent a VO_2max_ test on a treadmill. Oral health was assessed at their workplace on a separate occasion, no more than one week apart from the fitness assessment.

#### Ethical approval

Ethical approval was granted by the UCL Research Ethics Committee in the line with the Declaration of Helsinki (ID Number: 13985/004). The protocol was registered with ClinicalTrial.gov (Unique ID: NCT04204486) and has been reported according to the STrengthening the Reporting of OBservational studies (STROBE) statement [25].

### Participants

#### Recruitment

Participants were recruited via newsletters through their employers, and posters on announcement boards at the workplace throughout November and December 2019. Participants volunteered by contacting the lead researchers directly. The closing date for volunteering was 30 December 2019. A total of 89 participants were recruited. All participants signed an informed consent form before commencing the study. Participants with physical injury, neuromuscular, respiratory, or cardiovascular condition were excluded from the study. A complete data set of oral and periodontal data, and fitness data, were available for a total of 89 participants.

### Variables and data sources/measurements

#### Demographics

The following demographics variables were recorded: gender (male/female), age, ethnicity (1 = Black Asian, minority ethnic (BAME) 0 = other), time in service (0 = < 10 years; 1 = 10 to 20 years; 2 = > 20 years), sick days off in the last year (number of days), job performance (mean score) in the last 28 days before the starting of the study, use of pain killers (yes/no) and any medical conditions (yes/no).

Anthropometric measures included Height (cm), Weight (Kg), BMI, Muscle Mass (Kg) and Fat %. Body composition was analysed via bioelectrical impedance on a Tanita MC980MA (Tanita Cooperation, Tokyo, Japan) [26]. HbA1c levels were also measured by collecting one blood capillary sample with a finger prick. The capillary was then inserted in an Eppendorf tube and inserted into a Eurolyser Cube (Eurolyser, Salsburg, Austria).

#### Oral and periodontal health

Participants’ oral health was assessed on site at the respective stations in January and February 2020 through clinical examination.

Clinical measures of oral and periodontal variables were recorded using the same type of periodontal probe (UNC-15 probe) by the same operator (J.G.) and included:

- Total PPD > 4: Number of sites with probing pocket depth > 4 mm.
- % Sites with PPD > 4: percentage of sites with probing pocket depth > 4 mm.
- Total BoP Sites: Number of sites with positive bleeding on probing.
- FMBS: Full Mouth Bleeding Score.
- DMFT: Number of Decayed (DT), Missing (MT) and Filled Permanent Teeth (FT).

Periodontal pocket depth (PPD) was measured on 6 sites per tooth after accounting for missing teeth. The total number of sites with pocket > 4 mm over the total number of probed sites was expressed as percentage. Evidence from Needleman & Loos [27] on endpoints of periodontal therapy suggests PPD ≤ 4 mm is an indicator of disease stability. Bleeding on probing was recorded similarly to PPD at site level and then expressed as % bleeding sites over the total number of probed sites after accounting for missing teeth. For caries assessment, each tooth surface was examined and scored according to ICDAS criteria. Established caries threshold was ICDAS score 3 and above and reported at whole tooth level.

#### Physical fitness

Participants attended a physical screening session at the Institute of Sport, Exercise and Health (ISEH) at UCL. After recording anthropometric data, participants warmed up on a cycle ergometer at 60 rpm for 5 min, at a rate of perceived exertion of 6/10.

Cardiorespiratory fitness was measured using a Cardio-Pulmonary Exercise Test (CPET) via an incremental VO_2max_ assessment on a treadmill (h/p/cosmos, Nussdorf, Germany) using the Bruce protocol [28]. The protocol begins with 3 min walking (2.6 km/h) with no incline. Then every 3 min thereafter, the incline and speed of the treadmill are increased. Throughout the test, the participant is encouraged to continue exercising until volitional exhaustion, at which point the test is terminated, the treadmill returns to level at walking pace, the participant is instructed to walk slowly (2.6 km/h) and in silence for 3 min to recover fully. During the VO_2max_ test, Heart Rate was monitored via a *Polar* heart rate monitor, and breath-by-breath gas analysis was collected through a Vyntus CPX Metabolic Cart (Vyaire Medical, Chicago, USA). The anaerobic threshold was determined using the v-slope method, as the point of departure from linearity of carbon dioxide output (VCO_2_) plotted against oxygen uptake (VO_2_) [29].

For cardiorespiratory fitness, the following outcome measures were considered:

- Maximum load (Load_max_) (Watts) achieved during the Bruce treadmill test: this is a measure of performance and consists of the maximum load output the person generates at the end of an incremental test.
- Maximal oxygen uptake was measured by VO_2max_ (ml/kg/min): the maximal oxygen uptake is the highest VO_2_ achieved when a person is working at maximal capacity [30, 31].

Classically, VO_2_ reaches a plateau and does not increase further, even with an increase in external workload. Absolute values, which are typically expressed in litres per minute (L/min), may range from as low as 1.0 L/min (or lower in patients with cardiovascular disease) up to 6 L/min (or even higher in large, well-trained individuals). Because two individuals of quite different sizes may have the same absolute VO_2max_ value (mL/min), VO_2max_ is often normalised to body weight and expressed as mL/kg/min; this allows for between-person comparisons. Values for VO_2max_ typically range from a low of 10 (in clinical populations) to a high of 80 or more mL/kg/min in elite athletes, the average healthy and recreationally active adult will typically have a value around 35–45 mL/kg/min [32].

Lower limb power was measured via a countermovement jump (CMJ). The Countermovement Jump (CMJ) is a vertical jump test performed by squatting to a self-selected depth (typically about 90–70- degree external knee angle) and then jump as high as possible. It can be used to determine lower limb power, rate of force development, jump height and to measure lower limb asymmetries. Participants were instructed to place hands on hips, squat down and immediately jump up with straight legs maintaining hands on hips. Three CMJs were performed using a contact platform (Chronojump-Boscosystems, Bacelona, Spain) and the highest jump was recorded.

Outcomes of functional strength included:

- Countermovement jumps (CMJs) measured by CMJ Height Average (cm).
- Countermovement jumps (CMJs) measured by CMJ Power Average (W = Watts).

The equation used to convert CMJ (countermovement jump) performance into average power output (watts) was provided directly by the equipment’s software: Average power (W) = 21.2 × jump height (cm) + 23.0 × body mass (kg)– 1,393.

Upper body strength endurance was measured by:

- Press-ups in 60 s (N) measured as the number of press-ups completed by participants within a 60-second timeframe, performed with good form (e.g. not dipping the hips and using full range of motion).

Press-ups (or push-ups) are a widely recognized strength training exercise that primarily targets the muscles of the chest, shoulders, and triceps, while also engaging the core and lower body muscles. Participants were instructed to perform as many standard press-ups as possible within the allotted time of 60 s. A standard press-up was defined as starting from a plank position with hands placed slightly wider than shoulder-width apart, lowering the body until the chest nearly touches the ground, and then pushing back up to the starting position.

Levels of physical activity were also assessed by giving the participants wearable devices to measure their levels of physical activity during an 8-day period (full on and off shift cycle) before baseline data collection. Measures were recorded via an accelerometer worn on the non-dominant wrist (Actigraph, Pensacola, USA) and processed through the Actilife software. The software uses the two wearable devices to calculate proportions of the day spent in sedentary, light, moderate and vigorous activity. These measures were combined to show percentage of the day spent in each activity level.

### Sample size

A post-hoc power analysis was conducted using G*Power 3.1 to determine the required sample size for the multiple linear regression model. Based on a medium effect size (f^2^ = 0.15), a significance level of 0.05, and a desired power of 80% (1 − β = 0.80), the analysis indicated that a minimum of 97 participants was needed for a model with six predictors. The final study sample included 89 participants, which is slightly below the recommended threshold. However, the estimated R^2^ for all the models was approximately 0.50, indicating that these explained a substantial portion of the variance in the outcome variable. Given this strong explanatory power and the relatively small shortfall of 8 participants, the sample size is considered sufficient to detect medium-to-large-sized effects, and any potential reduction in power is likely to be minimal.

### Data analysis

#### Descriptive statistics

Descriptive statistics were expressed as mean and standard deviation for the quantitative variables and frequency and percentage for the qualitative ones. Description of variables and unit of measurements are reported above (Sect. 2.3).

#### Inferential statistics

Inferential statistics looking at the association between physical fitness and oral health were conducted using multivariable linear regression models. The explanatory variables included in each model were: “Age”, “Gender” (1 = male; 0 = female), “Fat %”, “% Sites with PPD > 4”, “FMBS” and “DMFT”. The following dependent variables were in turn used to construct the models: “Load_max_”, “VO_2max_”, “CMJ Height Average”, “CMJ Power Average”, “Press-ups in 60 seconds”; and proportions of the day spent in Sedentary, Light, and Moderate Activity measured by accelerometers.

For each separate analysis, the method of normality checks and Q - Q plots were applied to confirm that the data was sampled from a normal distribution. Linearity checks were carried out by plotting residual vs. predicted values.

Given the extensive range of variables in the original dataset (for full details, refer to the protocol registered on ClinicalTrials.gov, Unique ID: NCT04204486), a working set of variables based on defensible assumptions regarding the roles of the independent variables was generated. These assumptions were informed by prior studies and expert knowledge from our multidisciplinary research team [33]. Some variables were excluded a priori, as they were not directly pertinent to the study’s scope or our research question. An initial ‘global model’ was defined including variables known to be consistently associated with physical performance (functional strength) and cardiorespiratory fitness, such as gender and age. These variables were also tested for univariate significance across all presented models. Based on the explorative nature of the study, oral and periodontal variables were evaluated to assess potential associations with strength and cardiorespiratory markers, focusing on key components of periodontal status, including the extent and severity of disease (PPD), inflammation (FMBS), and oral health (DMFT). Backwards elimination regression method was implemented.

Estimated marginal means plots were also created. The level of significance was α = 0.05. The statistical analyses were carried out using JAMOVI Statistics Version 1.2.27.0.

## Results

### Descriptive statistics

Study population consisted of 89 subjects. Mean age was 41.5 years (± 8.3; [range 23–61]), 64 (71.9%) were male and 25 (28.1%) female, 63 (74.1%) were of any white background while 22 (25.9%) were of BAME.

Fourteen (17.1%) participants had been in law enforcement service for less than 10 years, while 60 (73.2%) for a period between 10 and 20, and 8 (9.8%) for more than 20 years, respectively. The mean time spent in service was 14.3 ± 5.7 years. Forty-one (47.7%) participants had a rota including night shift patterns, while 45 (52.3%) had only day shifts. Twenty-nine (34.1%) subjects reported some medical conditions and only 12 (14.1%) were considered relevant. A limited number of participants self-reported medical conditions potentially associated with periodontal status, including diabetes (1), rheumatoid arthritis (1), and hypertension (4). HBA1c levels were tested showing a mean value of 4.5 ± 0.6 (interquartile range: 4.2 to 4.9 with a minimum of 4 and a maximum of 5.9 excluding - the only 2 - outliers) which indicates an essentially non-diabetic study population. The mean fat % was 27.2 ± 6.5.

The mean DMFT score was 6.1 ± 5.0. The mean number of missing, decayed and filled teeth was 1.4 ± 1.7, 0.3 ± 1.0 and 4.4 ± 4.0, respectively. The mean percentage of sites with probing pocket depth > 4 mm was 1.0 ± 2.4 and the mean FMBS 7.2 ± 6.5.

Demographic characteristics, anthropometrics and summary of oral health variables are reported in Table 1.

**Table 1:** Demographic characteristics, anthropometrics and summary of oral health variables of the study population

Variable	Estimate
**Age**	
Mean (SD)	41.5 (8.3)
Range	23.0–61.0
**Gender**	
Female	25 (28.1%)
Male	64 (71.9%)
**Ethnicity**	
BAME	22 (25.9%)
Other	63 (74.1%)
**Weight (kg)**	
Mean (SD)	88.4 (18.3)
Range	51.7–159.7
**Height (cm)**	
Mean (SD)	175.5 (8.8)
Range	150.4–200
**BMI**	
Mean (SD)	28.4 (4.6)
Range	20.5–47.2
**Fat %**	
Mean (SD)	27.2 (6.5)
Range	13.8–43.3
**Muscle Mass (kg)**	
Mean (SD)	60.7 (11.6)
Range	34.2–88.1
**Waist Circumference (cm)**	
Mean (SD)	92.8 (11.9)
Range	62.3–126.4
**VO**_**2max**_ **(ml/kg/min)**	
Mean (SD)	34.6 (5.0)
Range	17.3–41.5
**Time in Service (years)**	
<10	14 (17.1%)
10 to 20	60 (73.2%)
>20	8 (9.8%)
**Shift Pattern**	
Day	45 (52.3%)
Night	41 (47.7%)
**DMFT**	
Mean (SD)	6.1 (5.0)
Range	0.0–20.0
**FMBS**	
Mean (SD)	7.2 (6.5)
Range	0.0–34.4
**% Sites with PPD > 4**	
Mean (SD)	1.0 (2.4)
Range	0.0–15.6

DMFT = Decayed, Missing and Filled Teeth; FMBS = Full-mouth Bleeding Score; PPD = Probing Pocket Depth; SD = Standard Deviation

### Inferential statistics

#### Maximum load (Loadmax) (W)

The mean score for Load_max_ was 336.2 ± 82.0 [range 155–615].

**Fig. 1:**
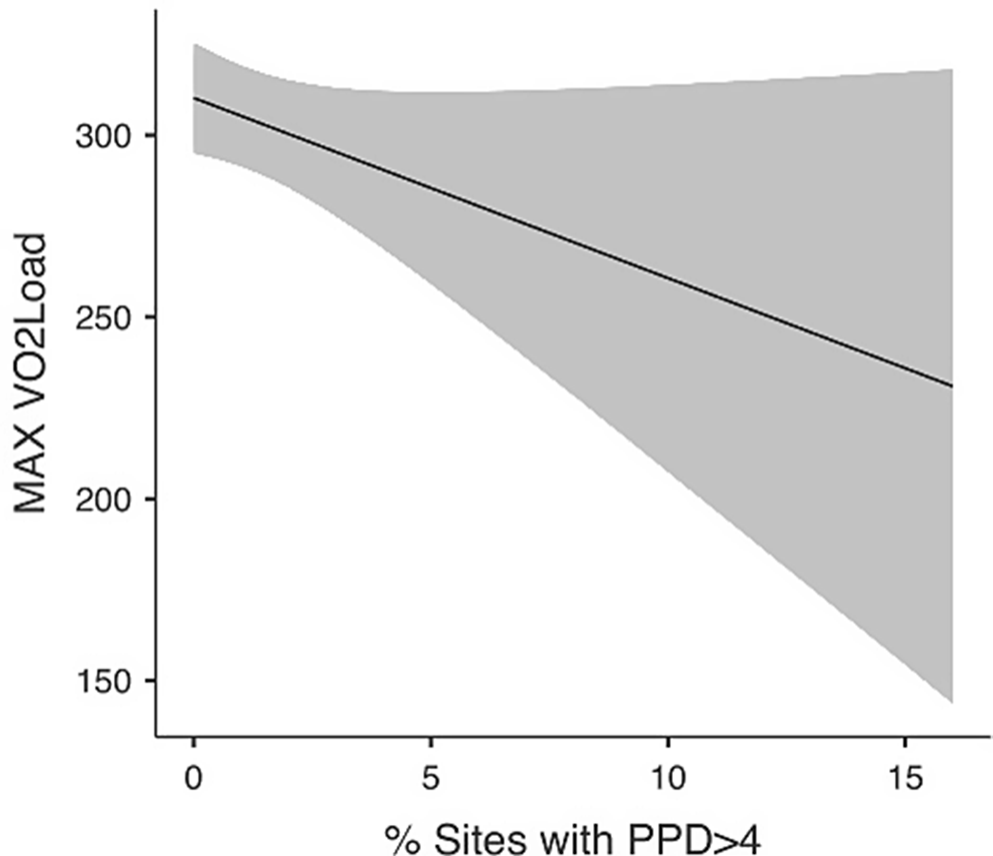
Estimated marginal means for Load_max_

Linear regression analysis revealed that Gender (Male) (β = 142.15; 95%CI = 111.14; 173.16; *p* < 0.001) and Fat % (β = 2.55; 95%CI = 0.38; 4.71; *p* = 0.022) were statistically significantly associated with increased Load_max_. Among the oral health variables, DMFT (β = 2.65; 95%CI = 0.15; 5.15; *p* = 0.038) was also positively associated with Load_max_ while the percentage of sites with probing pocket depth > 4 mm (β = -4.96; 95%CI = 10.74; 0.92; *p* = 0.092) was associated with lower Load_max_, though this relationship did not reach the statistically significant level. Age and FMBS showed no significant association with Load_max_. Results of the inferential analysis are reported in Table 2. Estimated marginal means plot for percentage of sites with probing pocket depth > 4 mm is reported in Fig. 1.

**Table 2:** Results of multivariable regression models for Load_max_, VO_2max_, CMJ height average, CMJ power average and press-ups in 60 s

Variables	Load_max_ (W) (β, 95% CI, *p*)	VO_2max_ (ml/kg/min) (β, 95% CI, *p*)	CMJ Height Average (cm) (β, 95% CI, *p*)	CMJ Power Average (W) (β, 95% CI, *p*)	Press-ups in 60 s (*N*) (β, 95% CI, *p*)
Intercept	188.848 (92.575, 285.12) < 0.001*	52.42 (45.60, 59.24) < 0.001*	32.37 (25.40, 39.34) < 0.001*	367.45 (161.67, 573.22) < 0.001*	36.52 (21.99, 51.04) < 0.001*
Gender (Male)	142.15 (111.14, 173.16) < 0.001*	-1.35 (-3.54, 0.85) 0.226	4.37 (2.08, 6.66) < 0.001*	433.66 (366.12, 501.19) < 0.001*	11.86 (7.09, 16.62) < 0.001*
Age	-0.95 (-2.44, 0.53) 0.206	-0.07 (-0.17, 0.04) 0.219	-0.10 (-0.21, 0.01), 0.068	-3.73 (-6.99, -0.47) 0.026*	-0.06 (-0.29, 0.17) 0.620
Fat %	2.55 (0.38, 4.71) 0.022*	-0.49 (-0.64, -0.33) < 0.001*	-0.34 (-0.50, -0.19) < 0.001*	13.05 (8.46, 17.64) < 0.001*	-0.86 (-1.18, -0.53) < 0.001*
DMFT	2.65 (0.15, 5.15) 0.038*	-0.08 (-0.26, 0.10) 0.374	-0.08 (-0.26, 0.10) 0.372	-0.05 (-5.27, 5.17) 0.985	-0.24 (-0.61, 0.13) 0.199
% Sites with PPD > 4	-4.96 (-10.74, 0.82) 0.092	0.06 (-0.35, 0.47) 0.786	-0.39 (-0.80, 0.02) 0.064	-8.99 (-21.15, 3.16) 0.144	-0.85 (-1.71, 0.01) 0.052
FMBS	0.58 (-1.59, 2.74) 0.596	-0.06 (-0.22, 0.09) 0.412	0.07 (-0.08, 0.23) 0.340	0.09 (-4.44, 4.61) 0.970	0.48 (0.16, 0.80) 0.004*

DMFT = Decayed, Missing and Filled Teeth; FMBS = Full-mouth Bleeding Score; PPD = Probing Pocket Depth; β = Estimated regression coefficient; 95% CI = 95% Confidence Interval; p = p-value; *statistically significant p-values

#### Maximal oxygen uptake (VO2max) (ml/kg/min)

The mean score for VO_2max_ was 34.61 ± 5.02 [range 17.3–41.5].

Regression analysis indicated that only body fat percentage was significantly associated with VO_2max_ (β = -0.49; 95% CI = -0.64, -0.33; *p* < 0.001), as no other covariates demonstrated significant associations (Table 2).

#### Countermovement jumps (CMJ) height average (cm)

The mean score for CMJ Height Average (cm) was 21.49 ± 5.54 [range 7.88–34.41].

Significant variables included being male (β = 4.38; 95% CI = 2.08, 6.66; *p* < 0.001) and body fat percentage (β = -0.34; 95% CI = -0.50, -0.19; *p* < 0.001). The percentage of sites with probing pocket depth > 4 mm (β = -0.39; 95%CI = -0.80; 0.02; *p* = 0.064) was associated with lower CMJ Height Average although the estimate did not reach the statistically significant level. Age, DMFT and FMBS were not associated with CMJ Height Average (Table 2).

#### Countermovement jumps (CMJ) power average (W)

The mean score for CMJ Power Average (W) was 868.6 ± 210.9 [range 393.7–1319.5].

The analysis indicated significant associations for gender (β = 433.66; 95% CI = 366.12, 501.19; *p* < 0.001) and body fat percentage (β = 13.05; 95% CI = 8.46, 17.64; *p* < 0.001), while no other variables reached statistical significance (Table 2).

#### Press-ups in 60 s (N)

The mean number for press-ups in 60 s was 20.14 ± 12.97 [range 0–53].

The regression model revealed that being male (β = 11.86; 95% CI = 7.09, 16.62; *p* < 0.001) and lower body fat percentage (β = -0.86; 95% CI = -1.18, -0.53; *p* < 0.001) were associated with a higher number of press-ups.

**Fig. 2:**
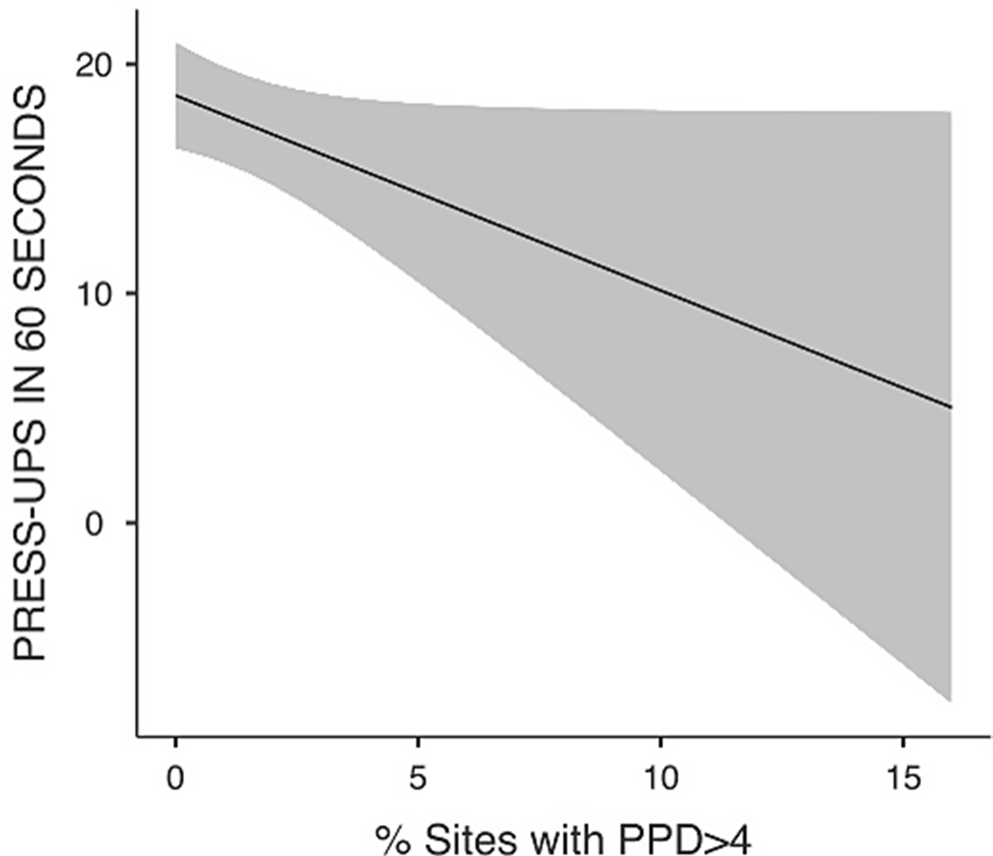
Estimated marginal means for press-ups in 60 s (N)

Additionally, the percentage of sites with probing pocket depth > 4 mm (β = -0.85; 95%CI = -1.71; 0.01; *p* = 0.052) was statistically significantly associated with lower number of press-ups in 60 s, while FMBS showed an opposite association (β = 0.48; 95%CI = 0.16; 0.79; *p* = 0.004). Age and DMFT did not show any statistically significant association (Table 2). Estimated marginal means plot for percentage of sites with probing pocket depth > 4 mm is reported in Fig. 2.

#### Levels of physical activity measured via accelerometers

The mean % of the day spent is sedentary, light and moderate activity was 41.44 ± 11.31 [range 25.18–63.71], 42.81 ± 10.17 [range 23.54–62.13] and 15.71 ± 4.62 [range 5.79–30.02], respectively. Results from the multivariable regression model results are presented in Table 3.

In the Sedentary Activity model, the intercept indicated that the baseline level of sedentary activity was 44.97 (95% CI: 17.92 to 72.02, *p* < 0.001). Gender showed a positive association suggesting that males may spend slightly more time in sedentary activities compared to females, although this finding was not statistically significant (β = 2.81; 95%CI = -5.52; 11.14; *p* = 0.500). Notably, FMBS was statistically significant (β = 0.78; 95%CI = 0.08; 1.48; *p* = 0.030), indicating that for every unit increase in the Full-mouth Bleeding Score, the proportion of time spent sedentary increases by 0.78%.

In the Light Activity model, the intercept was 39.87 (95%CI = 15.49; 64.25, *p* < 0.001), indicating the baseline time spent in light activity. A higher percentage of sites with probing pocket depth greater than 4 mm was associated with an increase in time spent in light activity (β = 1.33; 95%CI = 0.05; 2.61; *p* = 0.042). Conversely, an increase in the Full-mouth Bleeding Score was associated with a decrease in light activity time (β = -0.75; 95%CI = -1.37; -0.12; *p* = 0.022).

**Fig. 3:**
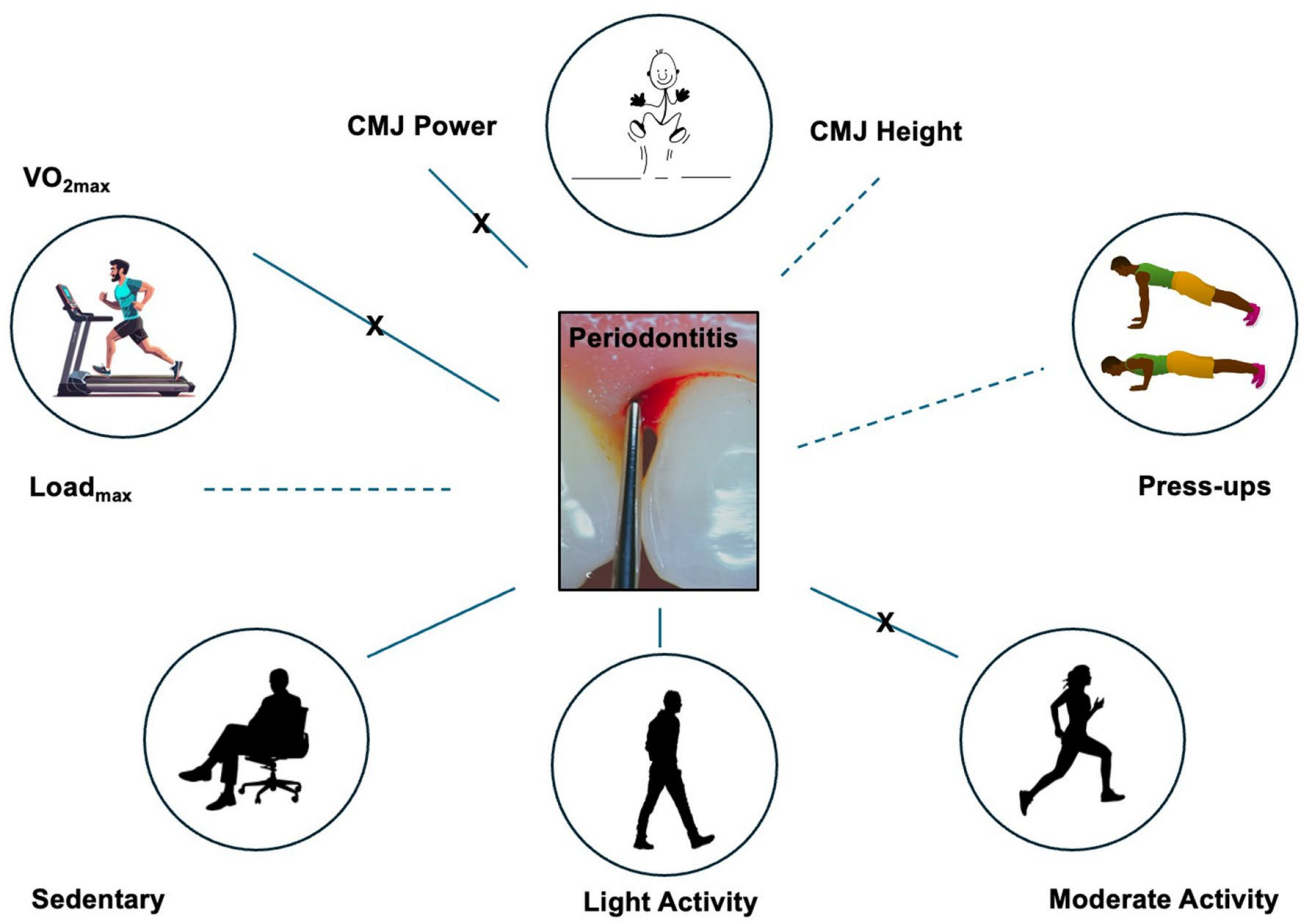
Periodontitis and physical fitness/performance outcomes. Line = statistically significant association; dotted line = trend/possible association (not statistically significant); crossed line = no association

In the Moderate Activity model, the intercept showed a baseline level of moderate activity at 15.66 (95% CI: 3.27 to 28.05, *p* = 0.020). None of the predictors in this model, including Gender, Age, Fat %, DMFT, % Sites with PPD > 4, and FMBS, demonstrated statistically significant relationships with the proportion of time spent in moderate activity (all p-values > 0.050). However, the coefficient for FMBS was slightly negative (β = -0.04; 95%CI = -0.36; -0.28; *p* = 0.778), suggesting a non-statistically significant association with the time spent in moderate activities. A diagram of estimated associations between periodontitis and physical fitness/performance outcomes is represented in Fig. 3.

**Table 3:** Results of multivariable regression models for proportions of the day spent in sedentary, light, and moderate activity

Variables	Sedentary Activity (β, 95% CI, *p*)	Light Activity (β, 95% CI, *p*)	Moderate Activity (β, 95% CI, *p*)
Intercept	44.97 (17.92, 72.02) <0.001*	39.87 (15.49, 64.25) <0.001*	15.66 (3.27, 28.05) 0.020*
Gender (Male)	2.81 (− 5.52, 11.14) 0.500	−1.23 (− 8.74, 6.29) 0.742	−1.57 (− 5.39, 2.24) 0.407
Age	0.06 (− 0.41, 0.52) 0.800	−0.09 (− 0.51, 0.33) 0.666	0.03 (− 0.19, 0.24) 0.800
Fat %	−0.38 (− 0.97, 0.22) 0.210	0.36 (− 0.18, 0.89) 0.185	0.01 (− 0.26, 0.29) 0.920
DMFT	−0.17 (− 0.82, 0.47) 0.590	0.16 (− 0.42, 0.75) 0.578	0.01 (− 0.29, 0.31) 0.951
% Sites with PPD > 4	−1.18 (− 2.60, 0.25) 0.102	1.33 (0.05, 2.61) 0.042*	−0.14 (− 0.79, 0.51) 0.668
FMBS	0.78 (0.08, 1.48) 0.030*	−0.75 (− 1.37, − 0.12) 0.022*	−0.04 (− 0.36, 0.28) 0.778

DMFT = Decayed, Missing and Filled Teeth; FMBS = Full-mouth Bleeding Score; PPD = Probing Pocket Depth; β = Estimated regression coefficient; 95% CI = 95% Confidence Interval; p = p-value; *statistically significant p-values

## Discussion

In this cross-sectional analysis of British law enforcement workers, periodontal disease extension and severity expressed by the percentage of sites with probing pocket depth > 4 mm were consistently associated with lower physical strength, including the maximum load output generated at the end of an incremental test and the power generated after countermovement jumps. Load is typically measured in Watts, where maximum load at the end of an incremental test can be used as a measure of maximal capacity, i.e., how much energy is needed to overcome inertia and create movement, like pedalling a bike, climbing stairs, powering the elliptical, running on a treadmill. Estimates obtained from the regression model suggested that a 10% increase in the number of sites with unstable periodontitis could be associated with an average 50 W lower maximum load on the treadmill (*p* = 0.092) following adjustment with covariates including age, gender and fat %. Considering that an average healthy adult will report about 300 W at peak performance on a treadmill test, a 50 W difference is a meaningful difference in peak performance, especially after having controlled for gender and fat % in the model. It should be noted, however, that heavier body weights require greater power, so an association with peak power output, in the absence of any association with VO_2max_ suggests that, in this specific cohort, periodontal health was associated with lower limb power but not CRF.

Vertical jumps, such as countermovement jumps (CMJs), are a simple and effective means to monitor power output from an individual [34, 35]. Our data suggested an average lower performance in CMJs of around 90 W in power (95% CI = -21.15, 3.16; *p* = 0.144) and 0.4 cm in height (95% CI = -0.80, 0.02; *p* = 0.064) for every 10% increase in sites with PPD > 4 mm. The average CMJ peak power output for this cohort was 897 ± 209 W, making a difference in 90 W a meaningful one. As with the treadmill test, these findings suggest an association with absolute lower limb power after adjusting for gender, age and fat%.

Of particular interest is the negative association observed between the percentage of sites with probing pocket depth (PPD) greater than 4 mm and the number of press-ups performed (β = -0.85; 95% CI = -1.71, 0.01; *p* = 0.052). Although this association did not reach statistical significance at the conventional level (*p* < 0.05), the trend suggests that poorer periodontal health, as indicated by increased PPD, may correlate with lower physical performance in upper body strength exercises. Similar findings were reported in a study of military police officers in Brazil [22]. Whilst the study employed a compound measure of ‘Physical Fitness Test’, three of the four components were related to muscle strength (push-ups, pull-ups and sit-ups). In relation to muscle strength alone, epidemiological data has demonstrated negative associations between periodontitis status and handgrip strength [36].

The results of the accelerometers analyses gave an insight on the possible relationships between various health-related factors, particularly indicators of gingival inflammation and daily physical activity levels. The positive coefficient for FMBS (β = 0.78; *p* = 0.03) is particularly notable, revealing that an increase in FMBS is associated with a significant rise in the proportion of time spent in sedentary activities suggesting that individuals with poorer oral health, as indicated by a higher FMBS, may be more inclined to engage in sedentary behaviours. Similarly, the negative relationship observed for FMBS (β = -0.75; *p* = 0.02) in the light activity model suggests that as oral inflammation increases, the time spent in light physical activities decreases. The negative association between periodontal inflammation and physical activity was also reported in a systematic review which found moderate quality evidence (GRADE) of an association between presence of periodontitis and a sedentary or inactive lifestyle across six studies involving 12,390 individuals [37].

The above results are in agreement overall with the current limited evidence from observational studies supporting a possible association between oral and periodontal health and physical fitness. A recent systematic review included 11 studies investigating the link of oral and periodontal health/disease status, malocclusion and peri-apical inflammation with objective measures of physical fitness [38]. Periodontal disease was evaluated in six studies (54.5%) and individuals who did not reach the highest physical fitness scores (measured by a variety of standardised physical fitness tests) presented significantly higher mean probing pocket depth (PPD) (*p* = 0.03), mean clinical attachment (CAL) (*p* = 0.01), mean bleeding on probing (BOP) (*p* = 0.04), and the number of teeth with CAL > 4 mm (*p* = 0.04) (Oliveira et al. 2015). In multiple regression models adjusted for age, body mass index (BMI) and waist-to-hip ratio (WHR), each mm of diminished periodontal attachment was associated with a reduction in handgrip strength (GS 0by 1.47 kg (95% CI -2.29 to -0.65) and 0.38 kg (-0.89 to 0.14) in men and women respectively [39]. However, to the best of our knowledge, none of the published studies has investigated the Load_max_, CMJs and press-ups outcome measures as reported in the present work.

Eberhard, Stiesch, Kerling, Bara, Eulert, Hilfiker-Kleiner, Hilfiker, Budde, Bauersachs, Kuck, Haverich, Melk and Tegtbur [20] reported that periodontitis was significantly associated with low measures of cardiorespiratory fitness (expressed as VO_2peak_) in sedentary men aged between 45 and 65 years. In our study, CRF evaluated through VO_2max_ did not show any significant association with oral and periodontal variables. This result is in agreement with the findings by Thai, Papapanou, Jacobs, Desvarieux and Demmer [21] on healthy young adults where no correlation between PPD and CAL with CRF was estimated. A possible common denominator between this and Thai’s study could be in the lack of relevant medical history in the participants of the present study.

FMBS and DMFT showed inconsistent direction of association across different outcomes. This could be explained by the generally low scores recorded in this sample (7% and 6%, respectively). Existing evidence suggests that tooth loss is independently associated with physical decline [40] and postural instability [41]. This could not be confirmed in the present investigation.

### Possible mechanisms

Maximum load (Load_max_) used in this study represents the peak power output generated by the participant at the endpoint of the treadmill test; to the best of our knowledge this outcome has not been reported before. Performance on an incremental treadmill test is mainly dependant on cardiovascular fitness. However, if a participant were to complete the test in non-optimal conditions (i.e. with fatigued muscles, being dehydrated, experiencing recent illness or inflammation etc.), their performance would be strongly impacted by their physical state, inducing a sense of fatigue earlier in the test, leading to exercise termination. Therefore, experiencing greater fatigue, regardless of fitness level, may lead to early test termination and thus a lower peak power output [42]. One of the proposed biological causes of fatigue suggests that an increased sensation of fatigue may be affected by higher levels of a polypeptide messenger molecule interleukin-6 (IL-6) during exercise. Periodontal disease has been associated to increased levels of cytokines [22], including IL-6 production which could in turn amplify the mechanism [43].

It may therefore be plausible that the increased systemic inflammation associated with periodontal disease [7] may in turn affect one’s engagement in exercise and performance on a maximal exercise test. Further research could investigate whether improving periodontal and systemic inflammation may optimise performance outcomes and exercise capacity.

Countermovement jumps (CMJs) take advantage of the stretch-shortening cycle to deliver power and sufficient body of evidence suggests that some of the negative effects associated with eccentric physical exercise including performance deficits may be related to elevated immune inflammatory response markers such as C-reactive protein (CRP) [44, 45], circulating lymphocytes and neutrophils [46] and elevations in inflammatory cytokines, including IL-1β, IL-6, tumor necrosis factor-α, and others [47–49]. These share similarities with systemic inflammatory changes induced as a result of periodontal disease [7]. Similarly to Load_max_, CMJ power analysis has not been reported before in correlation with exposure to oral and periodontal variables.

The extent of periodontitis was (almost) statistically significantly associated (*p* = 0.052) to the number of press-ups completed and these results were in line with those of a cross-sectional study [22] of male police officers, where individuals who reached the highest Physical Fitness Test (PFT), including push-up, pull-up, sit-up and running exercises had significantly better periodontal conditions compared with those with PFT scores below the maximum.

Poor oral health has been consistently associated with negative effects on self-reported performance in elite and professional athletes [50–53]. Pain was significantly associated with the performance effects along with specific psychosocial impacts arising from poor oral health as well as self-reported gum bleeding. Therefore, further mechanisms linking periodontal health with physical fitness could include pain (although not commonly a feature of periodontitis) and psychosocial factors including confidence and oral health-related quality of life.

Muscle power itself might have links with inflammation. In a recent study, Ogawa, Satomi-Kobayashi, Yoshida, Tsuboi, Komaki, Nanba, Izawa, Sakai, Akashi and Hirata [54] reported a significant correlation between oral health (number of teeth) and physical frailty and exercise capacity measured by a Short Physical Performance Battery (SPPB) (*r* = 0.24), grip strength (*r* = 0.33), and 6-minute walking distance (*r* = 0.26) in patients with CVD. Hand grip strength has also been associated with systemic inflammation [55], and higher levels of inflammatory markers and the clotting cascade have been found in frail persons compared with non-frail persons, where frailty was defined by unintentional weight loss, muscle weakness, slow walking speed, easy exhaustion, and low physical activity [56]. Although these findings were reported in clinical populations rather than a healthy group, this extreme association between inflammation and muscle weakness provides insight into a possible mechanism that may explain the relationship between strength and oral health in our cohort. It would be plausible that oral inflammation had a negative influence on this axis.

Other possible mechanisms explaining association pathways between periodontal diseases and physical fitness and performance outcomes may include metastatic pathways, as proposed for the biological mechanisms linking the chronic oral diseases and other chronic systemic diseases [38].

### Limitations

Several limitations can be attributed to this study. First, this was a cross-sectional study involving a specific population (British law enforcement officers) and therefore generalizability of the reported results may be limited. Moreover, the baseline periodontal assessment showed low full-mouth bleeding scores (< 10%) which would suggest low inflammation levels/active disease status. The % of sites with periodontal pockets was also limited indicating low disease prevalence. However, this could also be seen as a strength as despite this a consistent trend was found potentially indicating that stronger links may be seen in populations where periodontal disease extent and severity are greater.

Maximal oxygen uptake (VO_2max_) is widely accepted as the criterion measure of CRF. Association with the VO_2max_ outcome could not be confirmed in this study. While the lack of association with VO_2max_ is in agreement with Thai, Papapanou, Jacobs, Desvarieux and Demmer [21] who also analysed healthy individuals, the associations with lower limb power output on two separate assessments indicate that oral health might possibly be relevant to other components of performance. However, causality cannot be inferred from this data set.

A limited number of participants self-reported medical conditions potentially associated with periodontal status, including diabetes (1), rheumatoid arthritis (1), and hypertension (4). HBA1c levels were tested showing a mean value of 4.5 ± 0.6 (interquartile range: 4.2 to 4.9 with a minimum of 4 and a maximum of 5.9 excluding - the only 2 - outliers) which indicates an essentially non-diabetic study population. Data regarding smoking habits were unavailable. The small sample size and paucity of data restricted the ability to perform a comprehensive quantitative analysis of the relationship between these risk factors, oral health, and performance outcomes. Consequently, the results should be interpreted with caution.

### Future Research

Considering the limited number of studies published, further research is required to investigate the impact of oral and periodontal health on physical fitness, exercise performance and cardiorespiratory function. As the study had a cross-sectional design, inference on causal relationships between oral and periodontal health and physical fitness could not be established and the long-term effects remain to be assessed through inclusion of broader and more representative populations with higher severity/prevalence of periodontal diseases.

## Conclusions

Within the limitations of this study and the observational nature of the report, the results suggest a negative association between periodontitis and physical performance expressed as lower limb power, upper body strength endurance and proportion of time spent in physical activities. If these results are generalised to the wider population and confirmed by longitudinal studies, strategies on prevention and treatment of periodontal disease may assist in the optimisation of physical performance outcomes.

## Data Availability

The data that support the findings of this study are not openly available due to reasons of sensitivity. Data may be made available from the corresponding author upon reasonable request and with permission from third parties. No reference can be made in order to protect the anonymity of the organisation and of the officers involved.
